# Production and Use of Customizable Agarose Molds for Scaffold-Free Mouse Ovarian Follicle Culture

**DOI:** 10.3791/68871

**Published:** 2025-10-24

**Authors:** Hannes Campo, Prianka H. Hashim, Emily J. Zaniker-Gomez, Samuel Gauthier, Zihang Yan, Hao Zhang, James A. Ankrum, Francesca E. Duncan

**Affiliations:** 1 Department of Obstetrics and Gynecology, Feinberg School of Medicine, Northwestern University; 2 Department of Biomedical Engineering, Northwestern University; 3 Roy J. Carver Department of Biomedical Engineering, Pappajohn Biomedical Institute, University of Iowa

## Abstract

The ovarian follicle is the functional unit of the ovary that produces hormones and gametes needed to sustain female reproductive function and health. The ability to recapitulate folliculogenesis, ovulation, and luteinization *in vitro* has broad basic, translational, and clinical utility. The most advanced *in vitro* follicle growth systems maintain the follicle’s three-dimensional (3D) architecture, which is crucial for the development of meiotically competent metaphase II oocytes in humans. Recently, a scaffold-free method for *in vitro* follicle growth of mouse multilayer secondary follicles was developed and validated. For this, custom 3D printed molds were used to micropattern agarose with microwells that accommodate the volumetric expansion of follicles. Follicles grown in this scaffold-free environment showed comparable hormone production and viability relative to well-established alginate-based encapsulated *in vitro* follicle growth (eIVFG) systems. Importantly, agarose microwells are a scalable method, less technically demanding, and show improved follicle growth and ovulation rates relative to eIVFG. This methodology produces customizable molds that are biocompatible with the oocyte, a cell highly sensitive to material-specific leachates and other environmental contaminants. Further, follicles in this system are cultured in the same focal plane, enabling real-time timelapse imaging and analysis. To increase the accessibility of this new approach, this article details the methods needed to design and 3D-print master molds, create silicone molds for 24- or 96-well plates, and culture isolated multilayer secondary ovarian follicles in the agarose molds. This setup can also be integrated with a cost-effective time-lapse imaging system, enabling morphokinetic analysis. In addition, molds can be paraffin-embedded for downstream histological analyses. Overall, this user-friendly method is a versatile tool for follicle culture and can be customized further to promote the differentiation and maturation of germ cells within the context of the follicle to sustain complete *in vitro* gametogenesis.

## Introduction

The oocyte, or female gamete, contributes the bulk of the cytoplasm and half of the genetic material to the embryo at fertilization, which is essential for the development of the next generation. Oogenesis starts during embryonic development when primordial germ cells enter meiosis and arrest in prophase I. These oocytes reside within primordial follicles and constitute the ovarian reserve, a finite and nonrenewable pool from which they are progressively recruited. Follicles develop in a process known as folliculogenesis, with the goal of ultimately producing a mature gamete capable of fertilization^1, 2, 3, 4, 5^. Follicle development begins with an early, gonadotropin-independent phase where paracrine signaling is essential for the survival and growth of the follicle. As follicles mature to the secondary stage, they become reliant on gonadotropins, marking a shift to endocrine signaling. A key characteristic of secondary follicles is an oocyte surrounded by a minimum of two complete layers of cuboidal granulosa cells, along with the newly developed theca layer. Theca cells form the follicle’s outermost layer, providing androgens and structural support^6, 7^. Finally, a fluid-filled antral cavity forms and ruptures in response to a luteinizing hormone (LH) surge during ovulation, releasing a fertilization-competent gamete arrested at Meiosis II^2, 8, 9^.

The oocyte achieves developmental competence for fertilization and the ability to support early preimplantation embryo development, referred to as nuclear and cytoplasmic maturation, during oogenesis and folliculogenesis^10, 11, 12^. Cytoplasmic maturation is apparent morphologically, as oocytes expand throughout folliculogenesis. As a result of somatic cell proliferation, including theca, granulosa, and cumulus cells, follicles themselves experience significant volumetric expansion. In mice, the diameter of the follicle increases fivefold from early secondary to pre-ovulatory follicles. Bi-directional communication between the oocyte and surrounding somatic cells is essential for oogenesis. In primary follicles, the zona pellucida forms from proteins secreted by the oocyte, and communication between these follicle compartments is partly mediated by transzonal projections, which provide physical contact between these two follicle compartments^13, 14^. Hence, the follicular microenvironment is vital to correctly orchestrate the growth as well as nuclear and cytoplasmic maturation of the oocyte^2, 5^. To better understand the discrete mechanisms that support these processes, there is a need for advanced and physiologically relevant *in vitro* culture techniques. The ability to culture follicles *in vitro* has broad applications, including assisted reproductive technologies and fertility preservation^15, 16, 17, 18^, species conservation^19, 20^, drug development^21^, and toxicology screening^22, 23^.

*In vitro* follicle culture systems are also an essential intermediary step for the ability to generate gametes from pluripotent stem cells, also known as *in vitro* gametogenesis (IVG)^24^. Researchers have utilized embryonic stem cells, mesenchymal stem cells, and induced pluripotent stem cells to develop gametes in a variety of species, often relying on somatic cells to recreate the native follicle microenvironment^25, 26, 27, 28, 29, 30^. While major proof-of-concept milestones have been achieved through these methods, there is still a challenge of low-quality oocytes, reflected in low embryo development and live offspring rates following *in vitro* fertilization in mice^31, 32^. No live births have been achieved yet in other species, underscoring the need for continued refinement of protocols to fully support oogenesis and folliculogenesis.

Currently, several strategies are utilized for follicle culture. The technically least demanding and highest throughput approaches are scaffold-free methods, where follicles are placed in a plastic dish or membrane insert^33, 34^. Here, granulosa cells adhere to the culture surface, losing contact with the oocyte^33, 34^. The breakdown of the follicular architecture limits the usability and biological relevance of these techniques. However, scaffold-free culture in low attachment plates or inverted drops allows the follicle to maintain structural integrity in several mammalian species^35, 36, 37^. Unfortunately, with these techniques, it is difficult to control the interaction between follicles and the amount of mechanical support they receive. Hence, to better recapitulate *in vivo* physiology, culture methods have been developed where follicles are encapsulated in hydrogels with defined, tunable chemical compositions and/or mechanical properties^33, 34, 38, 39, 40, 41, 42, 43, 44, 45, 46, 47, 48, 49, 50, 51, 52, 53^ For example, the alginate-encapsulated *in vitro* follicle growth system can recapitulate key transcriptomic signatures observed *in vivo*^21^.

Despite the success and value of encapsulated culture protocols, there are several disadvantages. They can be technically challenging, laborious, low-throughput, and are not compatible with automatic imaging methods. Further, encapsulated culture methods need multi-step approaches for larger mammalian species due to the higher volumetric expansion of the follicle^37^. Moreover, encapsulated techniques exert uniform support in all directions, and follicles need to be released from the hydrogel prior to ovulation. This does not occur *in vivo*, where the follicle interacts with the surrounding ovarian microenvironment, which provides dynamic biomechanical support^54^. For example, the stiff ovarian cortex maintains quiescence of primordial follicles, while large growing follicles are typically found in the softer medullary compartment^55, 56^. Once a follicle with a competent oocyte has reached its terminal size, follicle wall and ovarian surface epithelium modifications allow for ovulation. Therefore, culture techniques where follicles are allowed to grow in one single direction with little resistance could better mimic *in vivo* physiology.

**Figure 1:**
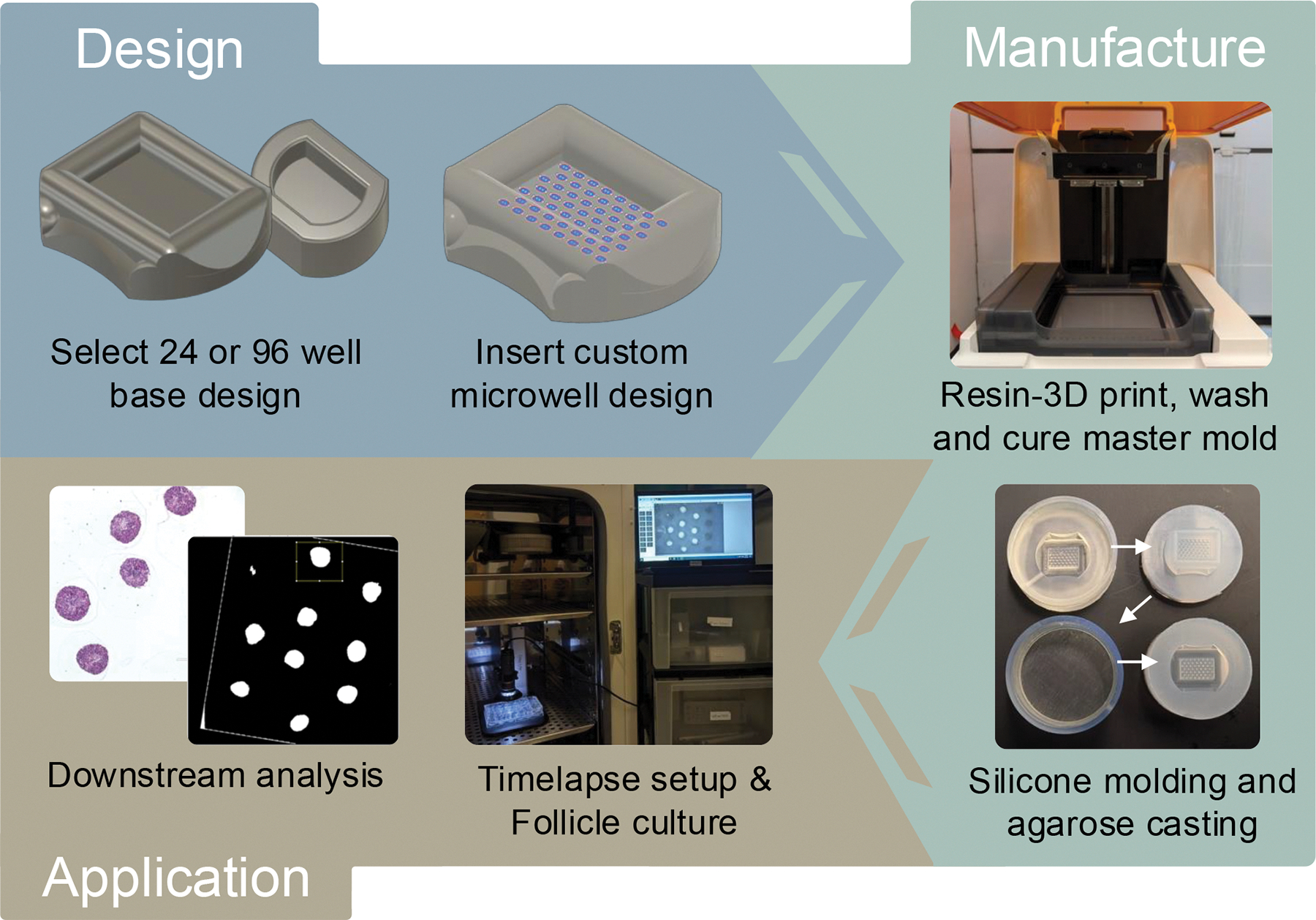
Overview of design, manufacturing, and application phases. A master mold is selected, the microwell design is incorporated, and the 3D files for the silicone containers are generated during the design phase. This is followed by the manufacturing phase, where designs are first 3D printed, washed, and cured. Then, the prints are inspected for defects and used during two silicone molding steps. Lastly, agarose molds are cast, equilibrated, and seeded with follicles within a timelapse setup. This scaffold-free culture method is compatible with downstream analysis using semi-automatic morphokinetic analysis, optical coherence tomography, and histological processing.

In a novel approach, a reproducible, scalable, technically less demanding, and cost-effective culture method for mouse multilayer secondary follicles was developed^57^. Here, ultra-low adhesion agarose microwells with precisely controllable geometry (shape, dimensions) and spatial arrangement (number, proximity) are manufactured using affordable methods. Follicles in the agarose molds experience mechanical support in all but one direction and are cultured in the same focal plane, enabling timelapse imaging for advanced morphokinetic analysis. Stereolithography (SLA) 3D printing was utilized as an approachable method to manufacture a master mold, followed by molding steps that create leachate-free silicone molds to cast the agarose. Improved follicle growth and ovulation outcomes were achieved using the new system compared to traditional eIVFG, without compromising overall follicle survival, maturation, or luteinization^57^. This protocol paper describes the steps necessary to customize and manufacture agarose molds, how to seed and culture multilayer secondary follicles, process, and finally perform timelapse analysis (Figure 1).

## Protocol

All animal procedures related to this protocol were approved by the Institutional Animal Care and Use Committee (IACUC) of Northwestern University and performed in accordance with the National Institutes of Health Guide for the Care and Use of Laboratory Animals.

### 1. Adapt master mold design

This section describes the first steps performed during the **Design** phase (Figure 1), where custom microwells are introduced in a computer-aided design (CAD) of a master mold for 24-well culture plates, called the **24-well master mold base design** (Supplemental File 1). The dimensions of the microwells dictate the amount of support and maximal growth size of follicles, while the number of wells determines the maximal number of follicles per agarose mold. The adapted master mold design will then be used in the next section to create biocompatible silicone molds for agarose casting.

**Figure 2:**
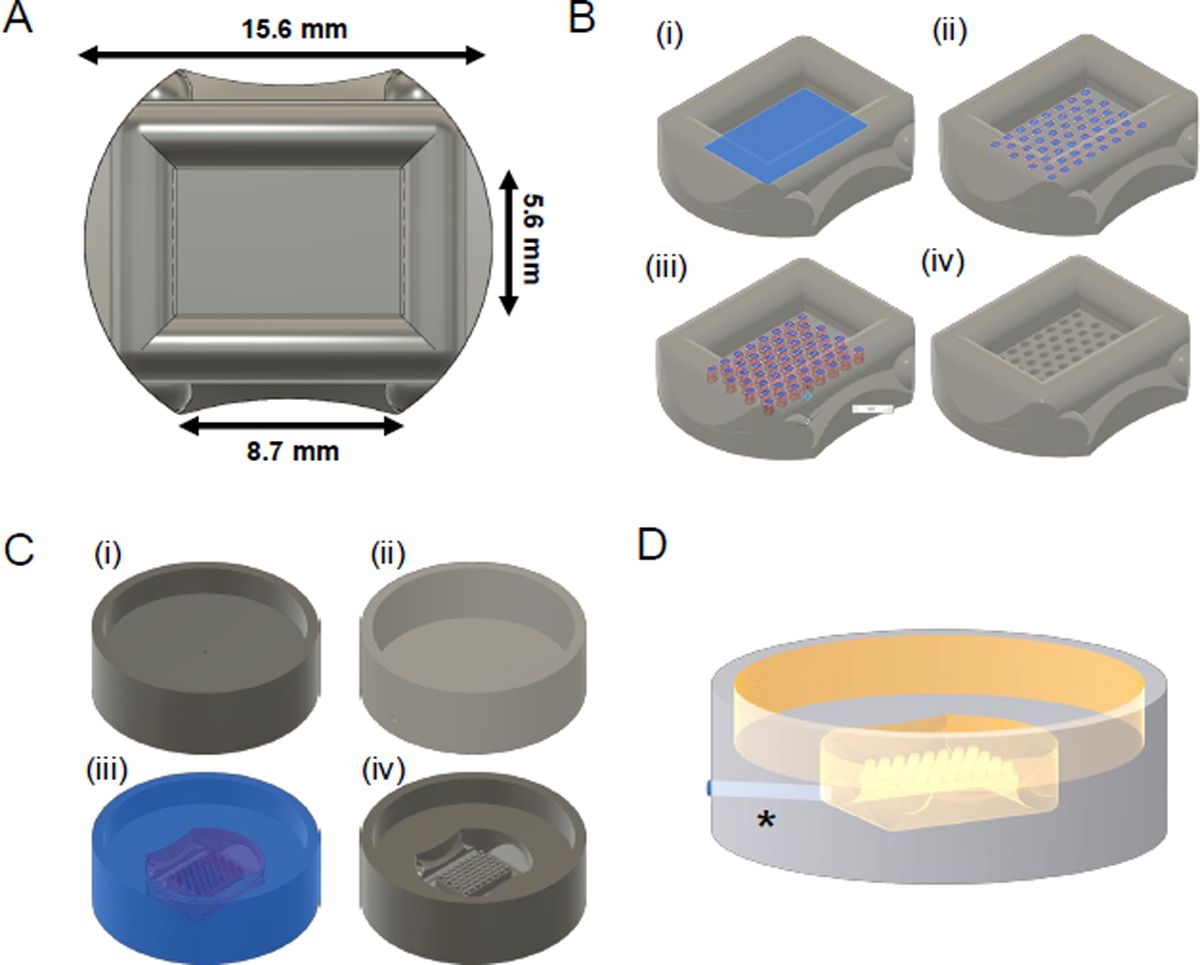
Preparation of computed assisted drawings for 3D printing. **(A)** Dimensions of 24-well plate master mold design. **(B)** Steps detailing the incorporation of custom micromold design, including (i) selection of the correct plane, (ii) two-dimensional microwell sketch, (iii) extrusion, (iv) and final adjustments. **(C)** Generation of silicone molding containers with base design of containers (i) 1 and (ii) 2, with the former to be modified to create the new master mold (iii and iv). **(D)** Bore channel (*) is incorporated using the print preparation software to avoid cupping during printing.

1. Open the **24-well master mold base design** .step file using CAD software (Figure 2A).
2. Select the internal surface area of the object, then navigate to the **Solid** tab in the **Design** workspace, and select **Create Sketch** to insert the desired micromold design (Figure 2Bi).
3. Introduce the desired xy dimensions and number of the microwells and select **finish sketch** on the toolbar (Figure 2Bii).
  **NOTE:** For multilayer secondary follicles, a 500 × 700 μm stadium geometric shape was used previously^57^. The printing resolution can vary depending on the resins and SLA printers used; allow for a minimal spacing of 400 μm between microwells.
4. Create the 800 μm deep microwells by using the **extrude** function (click on **Solid | Create | Extrude**). Make sure to select the **cut** operation, creating the microwell cavity (Figure 2Biii).
5. Select a **0.100 mm radius fillet** (click on **Design | Solid | Modify | Fillet**) for the top of the microwells
  **NOTE:** To create a round-bottom microwell, use the **fillet** operation again for the bottom of the culture well and have the **radius value** equal to the radius of the microwell design. For example, when designing 500 μm × 700 μm wells, select a 0.250 mm radius value (Figure 2Biv).
6. Export a copy of the **new master mold design** in .step format (click on **File | export**).

### 2. Creating silicone mold containers

**NOTE:** To prevent the leaching of cytotoxic compounds from the 3D printed parts, two biocompatible silicone molds will be made. This section describes the last steps in the **design** phase (Figure 1) and will result in the two containers used for silicone molding. Here, three different CAD files will be used, namely the **new master mold design** created from section 1, and **24-well silicone cast containers 1** and **2** (Figure 2Ci–ii and Supplemental File 2, Supplemental File 3, respectively).

1. Open the **24-well silicone cast container 1**.step file and insert the new master mold design as an external component (navigate to **Solid | Insert | Insert Component**).
  1. Right-click on the component and select **Break Link** to remove the linked reference to the new master mold design.
2. Center the **new master mold design** with **24-well silicone cast container 1** surface, making sure it faces inwards.
  1. Right-click the **new master mold design** object in the browser and select the **move/copy** option.
  2. Select the **Free Move** operation in the **Move Type section** and rotate the object 180° on the x-axis if necessary.
  3. Select the **Point to Point** operation in the **Move Type section** with the middle of the **new master mold design** object as the origin point and the center of the **24-well silicone cast container 1** surface as the target point.
  4. Select **OK** to align the objects.
3. Select the **cut** operation with the container as the target body and the master mold as the tool body design (Figure 2Ciii). This creates the container for the first step of silicone molding (Figure 2Civ).
  **NOTE:** The microwells that were designed in the first section are now micropillars that will be 3D printed as described in section 3. SLA printing is an additive manufacturing method; hence, printing micropillars improves print resolution compared to printing microwells.
4. Save and export the resulting **new 24-well silicone mold 1** as a .stl and .step file.

### 3. 3D printing of silicone containers

**NOTE:** This section describes the first steps performed during the **Manufacture** phase (Figure 1) where the new CAD designs with the desired microwell design features are 3D printed.

**Figure 3:**
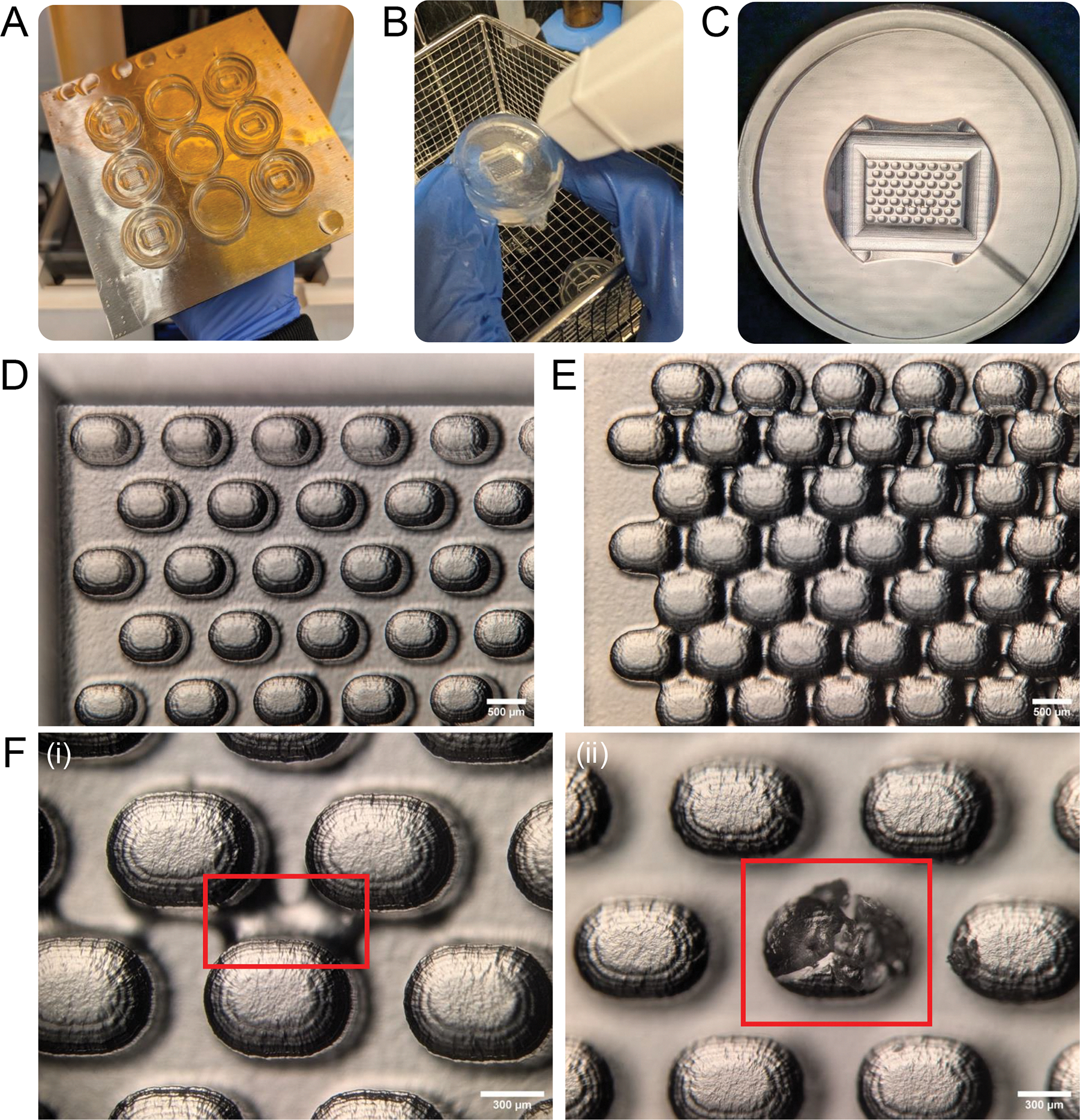
Resin-3D printing of silicone containers. **(A)** Silicone container designs are printed in triplicate. **(B)** Extensive washing of the master mold ensures efficient cleaning of important features of the print. **(C)** Resulting print viewed under stereomicroscope. **(D)** Example of correct printing of micropillars. **(E)** Example of micropillars separated less (300 μm) than the printing resolution allows. **(F)** Common printing defects such as bridges between (i) micropillars and (ii) damaged micropillars.

1. Open the .stl files of **new 24-well silicone mold 1** and **24-well silicone cast container 2** with 3D print preparation software.
2. Orient the print with the micropillars facing upwards and use the **drill hole** function to create a 1 mm wide opening (Figure 2D). Make sure this channel is open to the side of the print.
  **NOTE:** The bore hole that avoids cupping effects during printing and can be filled after printing, or the excess silicone material can be removed after the first silicone molding.
3. Print the two container designs at a 25 μm layer thickness (Figure 3A).
  **NOTE:** Print each model in triplicate to account for print imperfections or damage during processing.
4. Remove the molds from the printing platform and wash in 95% isopropanol according to the manufacturer’s instructions. Spray the micropillar section extensively with 95% isopropanol (Figure 3B). Remove any remaining ethanol with compressed air.
5. Let the remaining ethanol evaporate for at least 30 min.
  **NOTE:** At this stage, the 1 mm-wide drill channel can be sealed. This can be done by applying a small drop of unpolymerized resin with a p200 pipette tip on the outside of the print. Capillary forces will fill the channel.
6. Cure the 3D prints at 60 °C with ultraviolet light for 15 min.
7. Inspect each print using a stereomicroscope and discard any 3D prints with imperfections (Figure 3C–F). Thoroughly inspect every print visually, ensuring that all micropillars are separated and uniform in size and appearance (Figure 3D). Discard defect master molds with merged micropillars (Figure 3E).
  **NOTE:** Common issues are damaged micropillars or bridges from remaining unwashed resin (Figure 3Fi,ii).
8. Cover the outside of the print with parafilm if the drill channel is not filled and store until further use.

### 4. Silicone mold manufacturing

**NOTE:** This section describes the final steps of the **manufacture** phase (Figure 1), where two silicone molding steps will take place. This is necessary to prevent the release of toxic leachates by 3D printed parts.

**Figure 4:**
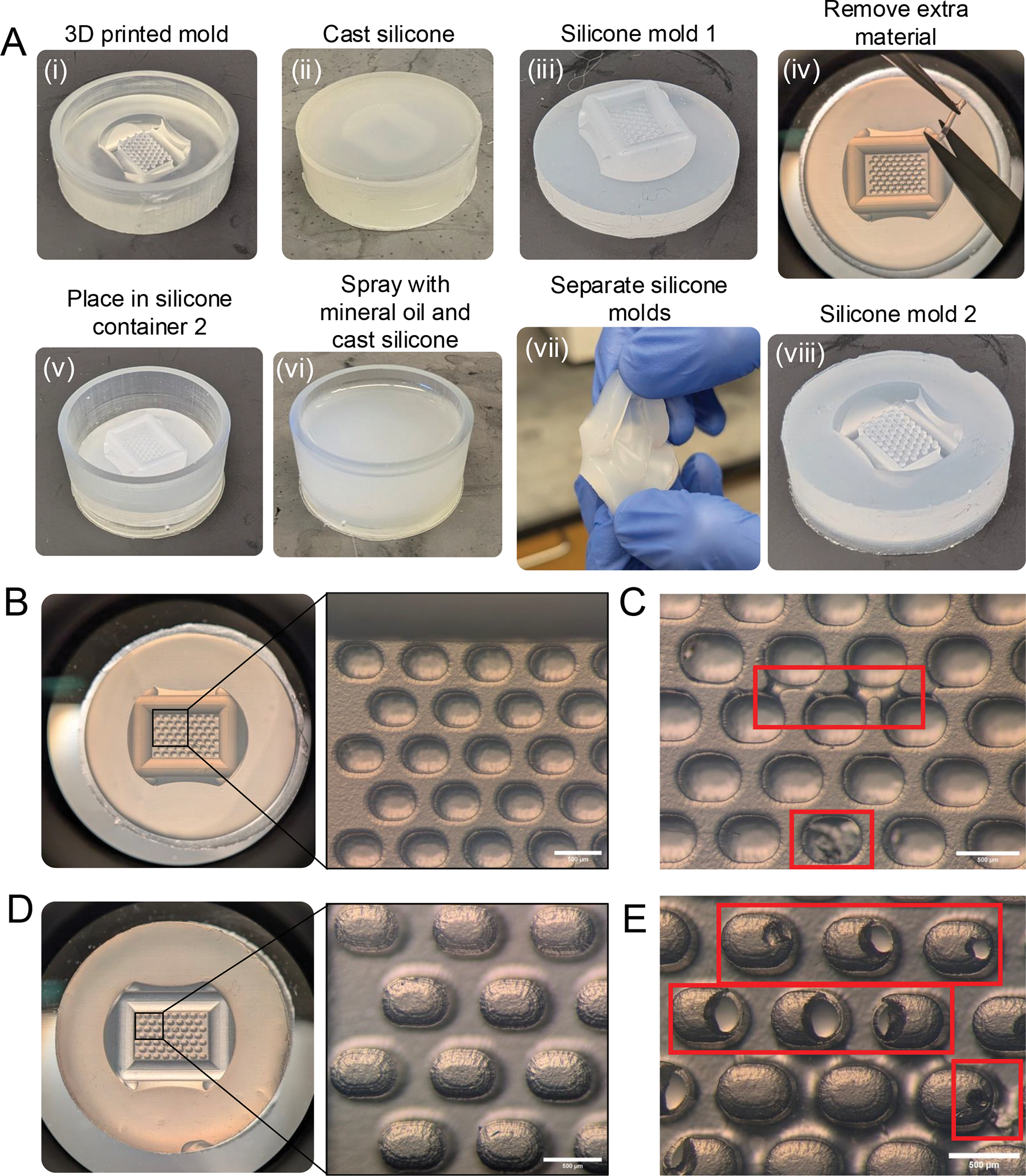
Manufacturing of silicone molds. **(A)** Steps performed during two silicone molding steps starting with the 3D printed master mold (container 1) until the final silicone mold 2 that will be used to cast agarose molds. **(B)** Inspection of silicone mold 1 using a stereomicroscope with examples of accurately molded microwells. **(C)** Examples of microwell imperfections due to bridging and compromised micropillars in the initial 3D print. **(D)** Inspection of silicone mold 2 using a stereomicroscope with examples of good micropillars. **(E)** Examples of imperfections of the micropillars due to unremoved microbubbles during silicone casting. Scale bars = 500 μm.

1. Prepare the silicone according to the manufacturer’s instructions, in summary:
  1. Mix the contents of **Part A** and **B** containers thoroughly, place a beaker on a scale and pour into container at a 1A:1B ratio by weight. Make sure to have excess material.
  2. Mix thoroughly for 3 min, making sure to scrape the sides and bottom.
  3. Place the material in a vacuum desiccator and leave for 5 min to eliminate trapped air bubbles. Repeat degassing up to 2x if air bubbles remain.
2. Pour the mixture with a uniform flow into the 3D-printed mold. Ensure that the mixture level is at the same level as the 3D print or slightly below to ensure flatness of the silicone mold (Figure 4Ai,ii).
  1. Remove any trapped air using a p200 pipette tip and use vacuum degassing if necessary.
3. Cure the silicone at room temperature for at least 5 h and preferably overnight before demolding.
4. Remove the silicone from the 3D printed mold and visually confirm the integrity of the micromolds using a stereomicroscope (Figure 4Aiii,B). Remove excess material from the drill channel if necessary (Figure 4Aiv). Discard molds with bridging or compromised microwells (Figure 4C).
5. Place the silicone mold in silicone mold container 2 for the second silicone molding step (Figure 4Av). Silicone container 2 has a 1 mm opening in the side wall that will be covered once the silicone mold is placed.
6. Lightly spray the silicone mold with embryo-safe mineral oil and remove any excess oil from the microwells. This will act as a release agent to separate both silicone parts after curing.
7. Repeat the mixing steps described in step 4.1 and pour the mixture into silicone mold container 2. Ensure that the mixture level is below or at the same height as the 3D print to ensure flatness of the silicone mold (Figure 4Avi).
8. Allow the silicone to cure at room temperature for at least 5 h, preferably overnight, before demolding.
9. Remove the silicone from the 3D printed mold and separate silicone molds 1 and 2 from each other (Figure 4Avii). Visually confirm the integrity of the micropillars using a stereomicroscope (Figure 4D). Discard the mold if bubbles or excess oil were present in the microwells (Figure 4E).
10. Wash the silicone mold with 70% ethanol and let air dry for 30 min in a laminar flow hood.
11. Place in a sterilization pouch and autoclave using a dry cycle to sterilize (Jacket pressure: 20 psi, Chamber temperature: 250 °F, Sterilizing time: 15 min).

### 5. Preparation of culture media

**NOTE:** Digestion of ovarian tissue for isolation and culture of follicles, *in vitro* maturation of oocytes, and luteinization requires six types of media: Dissection Media (DM), Enzymatic Media (EM), Maintenance Media (MM), Growth Media (GM), Maturation Media (IVM), and Luteinization Media (LM). Preparation of these media can be found as previously described by Converse et al.^38^ with an explanation of the importance of different media components analyzed by Simon et al.^33^, and their purpose is briefly mentioned in Table 1.

1. Prepare the media up to 1 week in advance and store at 4 °C.

### 6. Casting and storage of custom agarose mold and follicle culture

**NOTE:** This section describes how to cast custom agarose molds for follicle culture from the silicon molds generated in section 4. Agarose molds can be made and stored at 4 °C up to 2 weeks before follicle culture.

1. Place molds into a sterilization pouch and autoclave using a dry cycle as described in section 4.11. Allow the molds to cool down before use.
2. Make agarose solution by dissolving sterile 1.5% agarose (w/v) solution in Phosphate Buffered Saline (PBS) in a microwave or hot plate. Utilize a sterile conical tube that is placed in a beaker with water to allow for more even heating of the agarose and to slow down solidification of liquid agarose solution.
3. Pipette the sterile agarose solution into silicon casts in a laminar flow hood. Pipette slowly and smoothly to minimize bubbles from forming and ensure that the top of the mold is flat and not convex or concave. The top when casting will be the bottom of the mold when culturing and should lie flat. Use approximately 600 μL of agarose for a 24-well plate micromold and 85 μL for a micromold that fits in a 96-well plate.
4. Let micromolds cool for ~3 min until solidified. Invert silicon cast to expel micromolds onto a 100 mm Petri dish.
5. Assess the micromolds under a microscope for any deformities, including cracks, merging of microwells, or incomplete borders. Place the micromolds in sterile PBS + 1% Penicillin-Streptomycin and store at 4 °C for up to 2 weeks prior to use.
  **NOTE:** Make more molds than necessary to account for degradation over time or damage to micromolds when transferring between plates.
6. Equilibrate micromolds in 750 μL of Maintenance Media 2x for at least 1 h (total of two incubations) on the day of follicle isolation.
7. Remove all maintenance media, add 750 μL of Growth Media and place in incubator for 1 h.
8. Replace with fresh and equilibrated Growth Media prior to seeding.

### 7. Isolation and culture of late secondary follicles

**NOTE:** This section explains how to isolate and seed follicles in the agarose micromolds for follicle culture. Multilayer secondaries are cultured for 8 days, must have over 80% viability, and produce mature oocytes. Isolation and culture of secondary follicles have been described in significant detail elsewhere^38^. Isolation and selection of follicles for established alginate encapsulation and for seeding in agarose molds are consistent.

1. Dissect ovaries from mice and remove from periovarian adipose tissue and bursa. Place ovaries in 2.5 mL of Enzymatic Media and incubate in an orbital shaker at 37 °C for 20 min as previously described^57^.
2. Pipette 2 mL of Maintenance Media in an IVF dish and place in an incubator (37 °C, 5% CO_2_) prior to follicle isolation.
3. Quench reaction with 250 μL of FBS. Select for late secondary follicles after enzymatic isolation and transfer them to an IVF dish with DM.
4. Transfer follicles to an IVF dish with pre-equilibrated maintenance media and place in an incubator (37 °C, 5% CO_2_) for 1 h.
  **NOTE:** The starting size of late secondary follicles is 150–180 μm with more than two layers of granulosa cells present.
5. Add 750 μL of fresh GM to agarose micromolds and store in the incubator to prepare for seeding. Add sterile PBS to adjacent wells surrounding agarose micromolds to maintain humidity and minimize evaporation of media throughout culture period.
6. Seed follicles into agarose molds that have been pre-equilibrated and are submerged in GM. Transfer high-quality multilayer secondary follicles to agarose micromolds under a microscope utilizing a 200 μm stripper tip. Ensure that 10 follicles are transferred to each micromold in a 24-well plate and that each follicle is in a separate but adjacent microwell.
  1. Bend the stripper tip to allow for more precise manipulation and transfer of the follicles into the microwells.
  2. Transfer follicles quickly to minimize temperature and pH changes as MM and GM are CO_2_ buffered.
  3. Ensure that follicles within a micromold are of a similar starting size once all 10 follicles are transferred.
7. Place the seeded micromolds back into the incubator for at least 1 h to allow follicles to recover prior to imaging.
8. Image follicles at 4x and 10x magnification every other day to quantify follicle survival and growth outcomes.
  **NOTE:** Timelapse imaging can also be set up to perform morphokinetic measurements and are described in more detail in section 9.
9. Change 50% of culture media every other day.
10. Snap-freeze spent media with dry ice and then store at −80 °C. Use this conditioned media to assess hormone production through estradiol ELISAs.
  **NOTE:** If GM is made on the day of seeding (beginning of the culture period), the media can be stored at 4 °C and utilized for the entire culture period.
11. Remove and snap-freeze all growth media (750 μL) on day 8 of culture.
12. Replace growth media with IVM media to allow for *in vitro* ovulation.
  **NOTE:** Ovulation can also occur under timelapse imaging to assess timing and rupture features.
13. Remove cumulus-oocyte complexes (COCs) 14–16 h after IVM media has been added and score maturation status.
  1. Define the maturation status is as follows: identify MII by extrusion of the first polar body (expected outcome for healthy oocytes), GVBD/MI by the lack of a polar body or germinal vesicle (delayed maturation), GV by the spherical nucleus visible in the center of the oocyte (lack of meiotic progression) or degenerate-flattened and/or darkened oocyte.
14. **Optional**: As spindle parameters of MII oocytes, including chromosomal alignment, shape, and volume, are tightly correlated with oocyte quality and embryo outcomes^58, 59^, use them to assess the success of *in vitro* oogenesis. To assess spindle morphology, fix and stain MII eggs through whole-mount immunocytochemistry.
  1. Fixation: Fix the eggs in 3.8% PFA with 0.1% Triton X-100 at 37 °C for 20 min. Wash the eggs 3x in blocking buffer (PBS with calcium and magnesium (PBS +/+) with 10% Tween-20 and 0.3% BSA).
  2. Spindle staining:
    1. Incubate the eggs in permeabilization buffer (PBS +/+ with 0.1% Triton X-100 and 0.3% BSA) for 15 min at room temperature
    2. Rinse 2x in blocking buffer.
    3. Incubate the eggs in Alexa Fluor 488-conjugated anti-alpha-tubulin at a concentration of 1 μg/mL on a rocker wrapped in foil for 2 h at room temperature.
    4. Wash the eggs for 3 × 20 min in blocking buffer prior to mounting on microscope slides with DAPI-containing mounting medium.
    5. To observe the oocyte cytoskeleton, include 1:50 rhodamine phalloidin during staining.
  3. Image samples on a confocal microscope using a 63x objective with a z-stack at a width of 1.0 μm throughout the entire spindle.

### 8. Downstream applications of custom agarose molds

**NOTE:** Agarose micromolds can be utilized for histological analysis, allowing quantification and localization of different genes or proteins of interest. The micromolds create a follicle microarray that allows for multiple follicles to be stained in parallel. Optical coherence tomography (OCT) can also be performed without having to transfer follicles (Figure 1, Application). Optical Coherence Tomography is an imaging technique that allows for 3D reconstruction of samples and has higher penetration depth and resolution compared to confocal microscopy, allowing for improved imaging of 3D samples such as follicles cultured *in vitro*^60^.

1. For histology applications, make an agarose solution (1.5% w/v) as described in step 6.2.
  1. Remove all media surrounding agarose micromolds in the well and carefully remove media in the micromold cavity utilizing a p200 pipette at the corners of the cavity.
  2. Carefully pipette 100 μL of liquid agarose solution to fill and seal the microwells and cavity without agitating the follicles; ensure that the top is flat. Remove any bubbles with a sterile P10 pipette tip.
  3. Let the agarose solidify at room temperature for 2 min. Ensure under a microscope that the micromold is fully sealed.
  4. Fix the micromolds overnight at 4 °C with gentle rocking in 750 μL of fixative (Modified Davidson’s Solution or 3.8% paraformaldehyde).
  5. Aspirate the fixative and wash the sealed micromolds in ascending concentrations of ethanol, starting with 50% and finally 70% EtOH.
  6. Process using an automated tissue processer utilizing standard processing protocols (Table 2) and embed in paraffin wax. When embedding, make note of the orientation of the micromolds. Ensure that there is wax before and after the sample during the embedding process to minimize tissue loss during sectioning.
  7. Serially section embedded samples at 5 μm thickness and place on charged microscope slides.
  8. Perform H&E staining by an automatic stainer and seal with mounting medium prior to imaging.
2. When performing OCT, place the micromold with follicles in a 50 mm dish containing IVM media.
  1. To follow the procedure used to generate the representative OCT images, image using a custom-built OCT system operating within the visible-light spectral range with an axial resolution of 1.3 μm and a lateral resolution of 9.4 μm^61, 62^.
  2. Scan follicles in a 1.58 × 1,58 mm^2^ field of view, consisting of 512 A-lines and 512 B-scans, repeated 2x per B-scan and 3x per volume.
  3. Process OCT images using ImageJ to visualize the 3D structure of follicles and quantify volumes.
    1. Upload images into ImageJ as a Z-stack by dragging files into open application.
    2. Visualize the z-stack as a 3D image by going to **Plugins|3D|Volume Viewer**. Rotate the 3D image to view different angles at different time points.
    3. Navigate to **File| Save As |Tiff** to save the image to the desired location.

### 9. Timelapse analysis of follicle culture

**NOTE:** Follicles grown in the scaffold-free culture system reside in the same focal plane and are compatible with timelapse imaging in the incubator. Timelapse imaging can be started after seeding follicles in step 7.7 (Figure 1, Application).

Make sure that all changes are accurate throughout the timelapse. Hence, pass through the entire stack after every step to ensure there are no discrepancies in the measurements.

**Figure 5:**
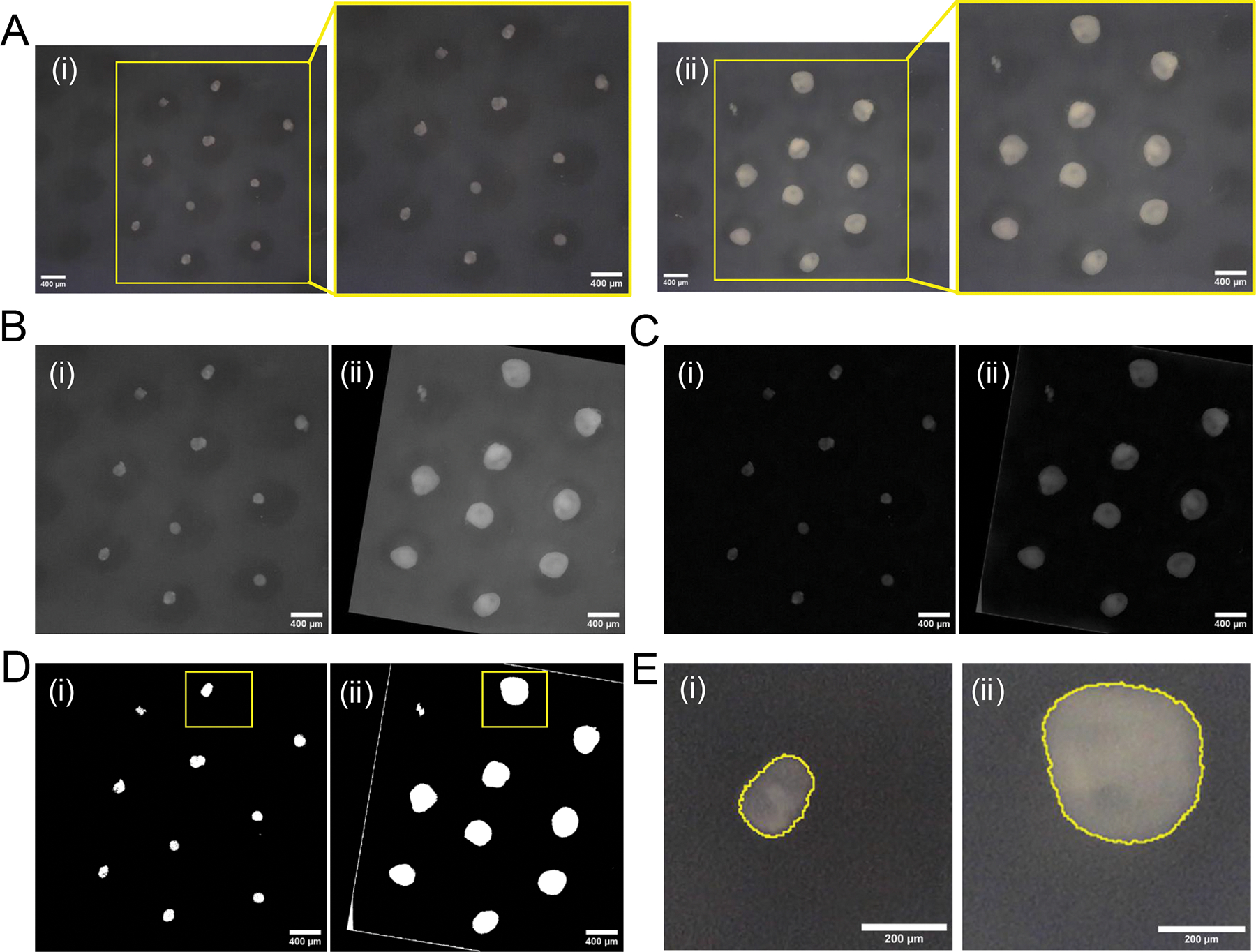
Timelapse analysis pipeline. **(A)** Cropping the initial stack to the smallest size possible, determined by the position of follicles between (i) the first and (ii) last images of the timelapse. Scale bar = 400 μm. **(B)** Representative images at day 0 (i) and day 8 (ii) after linear stack alignment with SIFT. Scale bar = 400 μm. **(C)** Rolling ball background subtraction of representative images at (i) day 0 and (ii) day 8. Scale bar = 400 μm. **(D)** Representative images at (i) day 0 and (ii) day 8 after thresholding. Scale bar = 40 0μm. (**E)** Particle analysis overlay for one follicle at (i) day 0 and (ii) day 8. Scale bar = 200 μm.

1. Timelapse setup:
  1. Place a Light focuser/concentrator cap on the handheld microscope.
  2. Put the handheld microscope with mount holder in the incubator and connect to a laptop with software installed.
  3. Place and align the culture well under the microscope and adjust the height and focus of the microscope so all follicles are in focus. Using Auto White Balance (AWB), choose optimal lighting conditions using **LED Control,** turn **Auto Exposure** (AE) **off**, and select the **optimal exposure time**.
    **NOTE:** Make sure that the alignment of the mold is the same before and after each media change; otherwise, analysis will have to be done separately for every 2 days of culture.
  4. Start timelapse imaging, selecting a duration of **8 days** and interval of **30 min**. Select **photo** and **Turn off LED when not taking pictures**.
Specify which measurements will be recorded in the set measurements dialog box (click **Analyze | set measurements**) before ImageJ analysis.
  **NOTE:** For more information on possible measurements incorporated into ImageJ software, see the user guide subsection 30.7^63^.
Place all images in a separate analysis folder to start analysis.
Drag and drop the analysis folder into ImageJ software to open the timelapse as a stack. Ensure that **sort names numerically** is selected and if image sequences do not fit in the RAM of the computer, select **virtual stack**.
Use the **rectangular selection** tool to select an area where all follicles throughout the timelapse are present. For this, use the last image first and scroll through the stack to make sure none of the follicles will be cut off. Then, crop the selection (navigate to **Image | Crop or Ctrl+Shift+X**, Figure 5Ai,ii).
Set the scale bar for the timelapse stack (**Analyze | Set Scale**). Utilize the day 0 image made with the imager and measure three identifiable features such as follicles or microwells. Use the same day 0 image from the timelapse stack and define the scale for one of the measurements; use the other two measurements to confirm the accurate scale is set.
Align all images with the linear stack alignment with SIFT plugin (Figure 5Bi,ii) using the following standard settings, which can be adjusted at a per case basis: **Linear Stack Alignment with SIFT, initial_gaussian_blur = 1.60 steps_per_scale_octave = 3 minimum_image_size = 64 maximum_image_size = 1024 feature_descriptor_size = 4 feature_descriptor_orientation_bins = 8 closest/next_closest_ratio = 0.92 maximal_alignment_error = 25 inlier_ratio = 0.05 expected_transformation=Rigid interpolate.**
Convert the stack to 8-bit grayscale (**Image | Type | 8 Bit**).
Remove the background utilizing the Rolling Ball Background Subtraction (click **Process | Subtract Background**, Figure 5Ci,ii) using the following settings, which can be adjusted at a per case basis: **Rolling ball radius=70.0 pixels**; select **disable smoothing**.
  1. Threshold the image (navigate to **Image | Adjust | Threshold**) to divide the timelapse images into two classes of pixels, using the following settings: **setThreshold(35, 255, “raw”); run(“Apply LUT”, “stack”); setOption(“BlackBackground”, true); run(“Convert to Mask”, “method=Intermodes background=Dark calculate black”)**.
Measure each follicle individually by using the rectangle selection, then select the **particle analysis** function (click **Analyze | Analyze Particles**). Use the following settings: Analyze Particles...“, “size= 8000-Infinity display exclude include summarize overlay add composite stack (Figure 5Di,ii).
  1. Before saving the results, confirm that the ROI for each image is accurate (Figure 5Ei,ii). Save Summary and Results in the .CSV format.
  2. Repeat this for all other follicles.

## Representative Results

**Figure 6:**
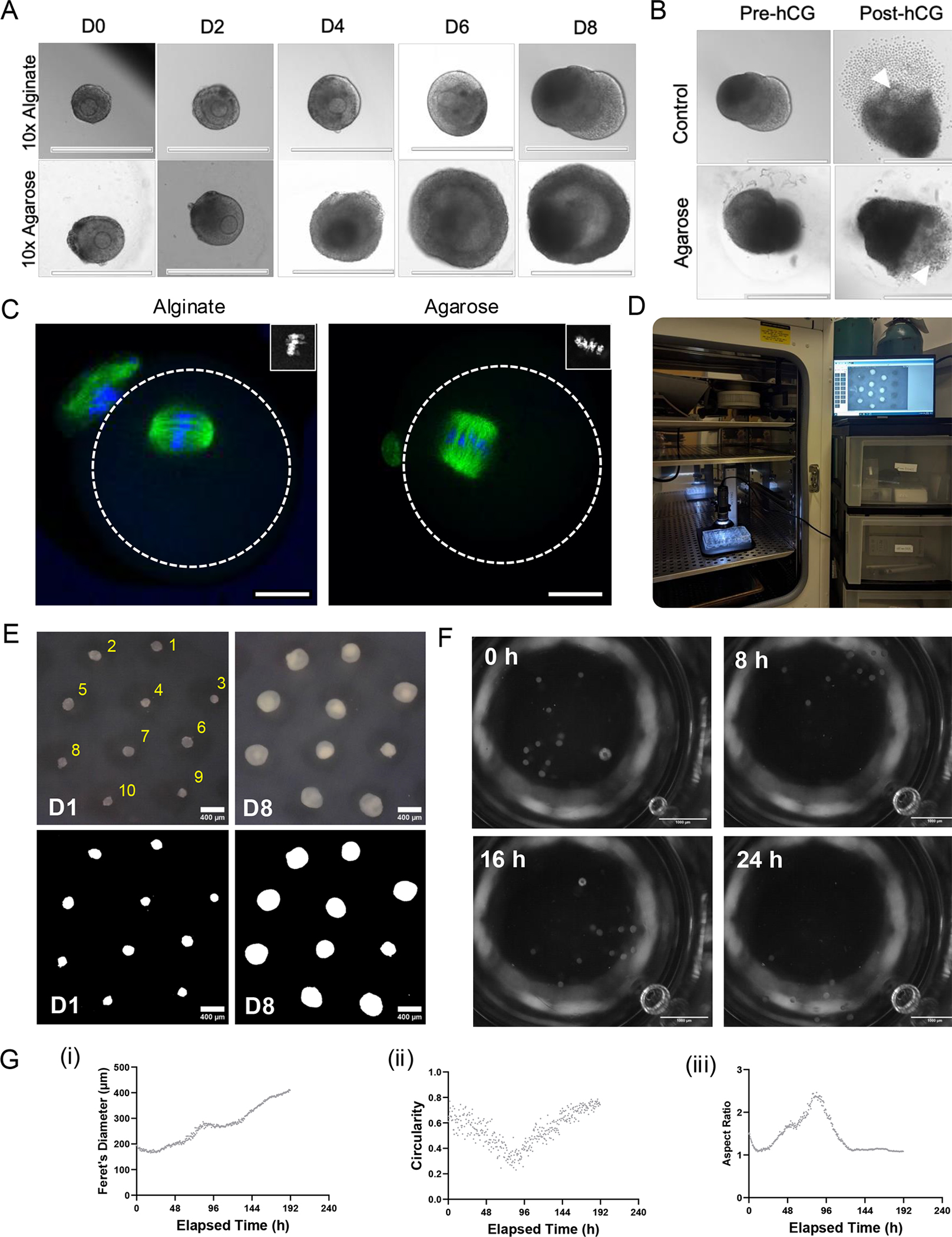
Key outcomes of secondary follicle culture and non-invasive monitoring of follicle growth kinetics. (**A**) Representative images of follicles cultured in alginate (top) or agarose micromold (bottom) across an 8-day culture period using a 10x objective. Scale bar = 400 μm. (**B**) Representative images of follicles prior to hCG (left) and post-hCG (right) exposure cultured in alginate (top) or agarose (bottom) using a 10x objective. Scale bar = 400 μm. (**C**) Representative normal meiotic spindles in MII oocytes from follicles cultured in alginate (left) or agarose (right). White circle outlines the borders of the egg. Green channel shows tubulin, and blue channel shows DNA using a 40x objective. Inset shows chromosomes. Scale bar = 25 μm. (**D**) Time-lapse culture setup within a standard incubator. (**E**) Alginate-embedded follicle movement over a 24 h period. Scale bar = 400 μm. (**F**) Representative images of cultured follicles (top) with automatically thresholded images used for quantification (bottom) at 70x magnification. Scale bar = 1,000 μm. (**G**) Example of other measurements that can be extracted for each follicle using this method; this includes (i) Feret’s diameter, (ii) circularity, and (iii) aspect ratio, amongst others.

To determine whether follicle survival and growth were supported using this novel technology as described in protocol section 7, follicles from the same mice were isolated and cultured in parallel in agarose micromolds or encapsulated in 0.5% alginate. Representative images of follicles from every other day of culture demonstrated normal development, growth, and formation of an antral cavity throughout the culture period (Figure 6A). Previously published results demonstrated that follicle survival shows no significant differences between culture methods and a significantly larger diameter for follicles cultured in agarose^57^. This indicates that agarose micromolds support folliculogenesis at an improved rate compared to alginate encapsulation.

Meiotic progression and spindle alignment of oocytes from follicles cultured in agarose micromolds were assessed. Ovulation was induced for follicles in both conditions as described in protocol step 7.11. Follicles that survived on Day 8 underwent *in vitro* ovulation, and representative images prior to (pre-hCG) and post (post-hCG) ovulation are shown for both conditions (Figure 6B). Previous experiments indicated that follicles cultured in agarose had a significantly higher percentage of ovulated eggs compared to alginate. Of the eggs that were ovulated, there was no significant difference in the percentage of MII eggs between conditions^57^. To determine spindle morphology, MII oocytes were fixed and stained with alpha-tubulin as described in protocol step 7.14. Bipolar spindles with chromosomes tightly aligned on the metaphase plate are considered normal. Multipolar or monopolar spindles and/or the presence of misaligned or scattered chromosomes were considered abnormal. Representative images of normal spindles cultured in scaffold-free and encapsulated conditions are shown in Figure 6C. The percentage of normal and abnormal spindles in MII eggs showed no significant differences between eggs from follicles cultured in agarose or alginate. These results indicate that there are improved numbers of ovulated eggs, follicle size, and no negative impact on meiotic competence or spindle morphology for follicles seeded in agarose and cultured scaffold-free.

This culture method is compatible with timelapse imaging because all follicles grow in the same focal plane during culture. With a simple setup utilizing handheld microscopes and mounts, all follicles can be clearly imaged throughout culture, and morphokinetic growth parameters can be extracted (Figure 6D,E). Unfortunately, alginate-encapsulated follicles do not maintain their position during culture and are incompatible with timelapse imaging (Figure 6F and Supplemental Video S1). Results indicate that follicle growth is not uniform within one mold, with some follicles still in an exponential growth phase, plateauing and others reducing in size at day 8 of culture^57^. Figure 6Gi–iii provides some examples of other dimensional variables that can be measured for each individual follicle, including Feret’s Diameter, circularity, and the aspect ratio of the major axis over the minor axis. These morphological parameters allow for the analysis of the longest diameter, roundness and elongation of the follicles during culture, respectively.

**Figure 7:**
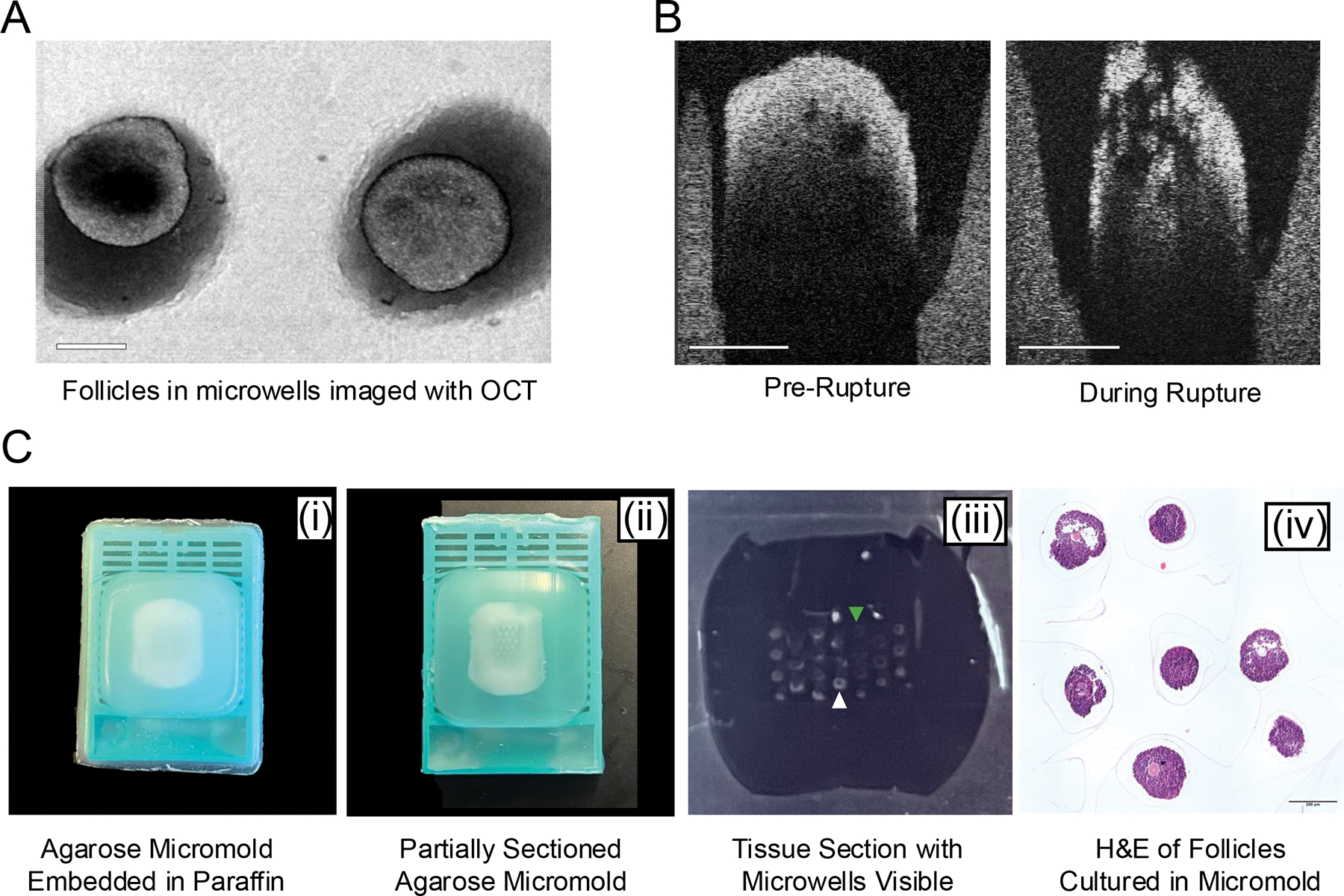
Downstream analysis. **(A)** Representative images of follicles cultured in agarose micromolds and imaged with optical coherence tomography. Scale bar = 200 μm. **(B)** Representative images of ovulation at early (left) and late (right) timepoints. Scale bar = 200 μm. **(C)** Representative images of utilizing agarose micromolds for histology. Agarose micromold embedded in paraffin (i) before and (ii) after sectioning. (iii) A tissue section shows visible microwells with (white arrow) and without (green arrow) tissue. (iv) H&E image of follicles sectioned and stained in an agarose micromold. Scale bar = 200 μm. Abbreviations: OCT = optical coherence tomography; H&E = hematoxylin-eosin.

Similarly, this method is also compatible with advanced high-resolution imaging techniques, including OCT. With OCT, 3D images with full depth penetration can be taken of follicles within the micromolds (Figure 7A). Follicles can then be viewed at cross sections to visualize internal structures such as the antral cavity and the follicle wall thickness (Figure 7B). OCT can also be used to visualize the directionality of follicle rupture by imaging an individual follicle at multiple time points during ovulation (Figure 7B). Agarose micromolds can also be utilized as a tissue microarray, where multiple follicles can be sectioned in the same plane for histological analysis as described in protocol step 8.1. Capped agarose micromolds can be processed using the same settings as whole tissue and embedded with specific orientation (Figure 7Ci). When serially sectioned, the microwells become visible (Figure 7Cii), and more care should be taken to not damage follicles through the sectioning process. Paraffin sections with visible microwells (Figure 7Ciii) can be assessed to determine whether tissue is present (white arrowhead) or absent (green arrowhead). Tissue sections with multiple follicles in the same plane can then undergo H&E staining (Figure 7Civ) or other histological analysis, such as immunohistochemistry, which is not possible when follicles are encapsulated, fixed, and sectioned in a hydrogel.

**Figure 8:**
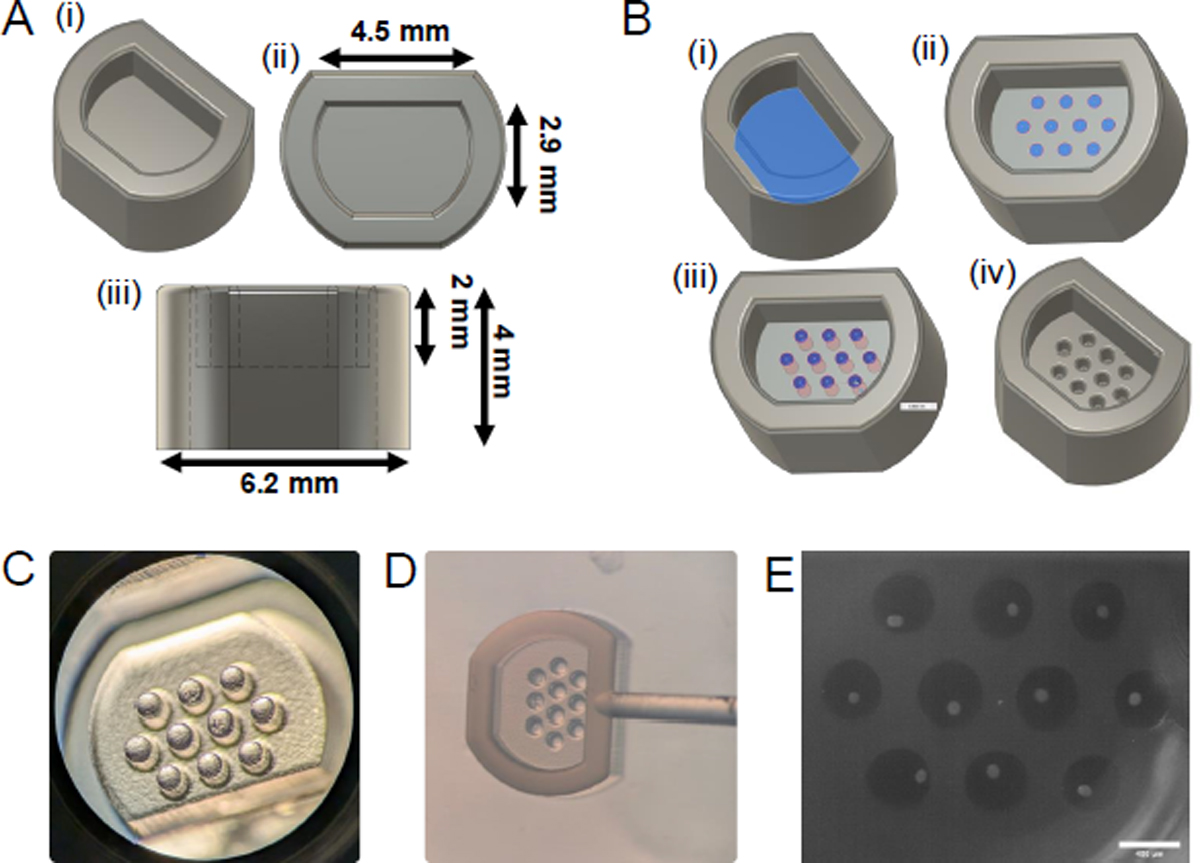
Mold designs for a 96-well plate. **(A)** Dimensions of 96-well plate master mold design. **(B)** Steps detailing the incorporation of custom micromold design, including (i) selection of correct plane, (ii) two-dimensional microwell sketch, (iii) extrusion, and (iv) final adjustments. **(C)** Resulting 3D-print viewed under stereomicroscope and **(D)** example of silicone mold 1. **(E)** Example of ten early secondary follicles (average diameter 115 μm) seeded in round 400 μm micromolds. Scale bar = 400 μm.

Lasty, to reduce culture media use and increase experimental output, this methodology can be applied towards smaller culture inserts. A master mold specifically designed for 96-well plates was included and can be created using the same methodologies (Figure 8Ai–iii, Supplemental File 4, Supplemental File 5, and Supplemental File 6). Microwell designs can be incorporated as described in protocol section 1 and adapted to the requirements of follicles of any stage (Figure 8B). Using the steps described in protocol sections 3 and 4, this design can be 3D-printed and used to create silicone microwells (Figure 8C,D). This design is compatible with timelapse imaging and other downstream analysis described in this protocol paper can be adapted towards this use as well (Figure 8E).

## Discussion

*In vitro* gametogenesis and maturation are key technologies for fundamental understanding of oocyte biology, with potential applications in assisted reproductive technology, fertility preservation, and species conservation. The methods to support IVG often rely on co-culture with somatic cells to create reconstructed ovaries^30^. The importance of these support cells is well-known *in vivo,* where the follicular somatic microenvironment is critical to healthy oocyte growth and development^2, 3, 8, 9^. Unfortunately, current *in vitro* follicle culture methods generate oocytes with decreased activation, negatively impacting fertilization and embryo development compared to *in vivo* gametes, with different transcriptomic signatures^64, 65, 66^. The reported efficiency of blastocyst production from *in vitro* culture is typically 10–20% of all eggs fertilized, compared to >60% from mature eggs collected *in vivo*^47, 67, 68^. Hence, follicle culture technologies that better mimic *in vivo* physiology could encompass key intermediary steps to improve IVG technique outcomes.

This protocol details how to design, manufacture, and apply agarose molds with microwells of defined geometries for scaffold-free follicle culture (Figure 1). Several steps are crucial to the protocol’s success. First, design features should not exceed the printing resolution of the SLA 3D printer, and intricate microwell geometries and spacings should be tested first by performing test prints. If a microwell depth larger than 800 μm is required, approximately 1 mm must be maintained between the bottom of the well and the bottom of the mold to ensure overall mold integrity. The manufacturing phase requires extensive visual inspection of the micropillars after the 3D printing and silicone pouring steps. Minor defects to the micropillars and microwells (Figure 3F, Figure 4C, and Figure 4E) can be introduced at every manufacturing stage, and defective molds must be identified and removed so as not to cause problems with the culture steps. Further, when utilizing a releasing agent to separate the two silicone molds in protocol step 4.6, be sure to only use embryo-safe mineral oil as biocompatibility of the final product could be affected otherwise. When seeding follicles, it is important that they are of the correct stage, have a similar starting size and healthy morphology, and are seeded in adjacent culture wells to optimize paracrine signaling. A detailed description of these stringent follicle selection criteria has been previously reviewed^33, 38^, and these should be followed when performing any type of follicle culture. Multilayer secondary follicles were isolated from prepubertal CD-1 mice for this study due to the high yield relative to adult animals. The ovarian reserve varies across mouse strains, which will impact the yield of follicles and follicle size^69, 70^. There is also a decrease in the number and quality of follicles with age^38, 71^. In fact, eIVFG using follicles isolated from older mice exhibits decreased follicle survival and growth compared to younger counterparts^38, 71, 72, 73^. Given that the ovarian stroma from older mice is fibrotic and stiff, enzyme-based isolation methods may be necessary with increased incubation time or higher enzyme concentrations^74, 75, 76^. Lastly, agarose concentrations and microwell dimensions may need to be optimized to better support follicles from other species. For example, activated preantral follicles from mice cultured in stiffer alginate (2% compared to 0.5%) showed decreased follicle survival and growth throughout 10 days of culture. Within 3 h of encapsulation, follicles cultured in 2% alginate exhibited downregulation of pathways associated with development and water homeostasis compared to follicles cultured in 0.5% alginate, indicating high sensitivity of follicles to the ovarian microenvironment^77^. However, bovine primordial follicles cultured in 1% or 5% alginate did not have significantly different growth outcomes throughout the culture period^78^. Therefore, further optimization will likely be required to successfully apply this technology to different follicle stages, or follicles from different species or across ages. Of note, if the incidence of follicle survival in any given experiment is less than 80%, it should be excluded from further analyses, and best practices for follicle culture should be revisited^33, 38^.

One of the main advantages of the agarose molds is their customizability. While ultra-low attachment microwells can be purchased commercially, they have set microwell designs and do not provide the same control over well dimensions, geometry, number, and proximity necessary for follicles to survive throughout culture^79^. It is expected that the molds can be adapted to support follicles of earlier stages, additional species, or from older donors, all of which are known to have different follicle survival and growth outcomes^73^. Furthermore, reconstructed ovaries (rOvary) made from pluripotent stem cells require co-culture with ovarian somatic cells in low-adhesion U-bottomed plates^31, 80^. It is possible that the agarose molds, after microwell design iterations and optimizations, could support the creation of multiple rOvaries in one culture well, mimicking *in vivo* cell signaling. However, additional studies confirming proper follicle survival, growth, and oocyte quality will be necessary to validate this technology for these proposed applications^33^.

This customizability is enabled by SLA 3D printing, an accessible and precise 3D printing method that allows for rapid iterative design improvement. Further, by including two silicone molding steps avoids reproductive toxicity concerns associated with resin-3D printed materials^81, 82^. Meiotic maturation is a highly sensitive process, and perturbations due to various factors, including environmental exposures, can result in reduced meiotic resumption and progression, defective spindle formation, and/or abnormal chromosome alignment. As such, biocompatibility is essential for the formation of healthy, mature eggs. Thus, follicle culture is inherently a highly sensitive biological assay to demonstrate cytotoxicity. Previous work has demonstrated that leachates from 3D-printed plastics induce oocyte degeneration and spindle abnormalities, which can be avoided by including silicone molding steps^57, 81^. This means that customizations can be made without concern of the toxicity on the gamete, one of the most sensitive and long-lived cells in the body^83^. Importantly, the custom agarose molds lower the technical barrier to culture follicles in a physiologically relevant environment. The scaffold-free agarose micromolds confine the follicle in all directions but one, mimicking permissive growth to prepare for ovulation found *in vivo*. This differs from eIVFG where the follicle experiences uniform mechanical stimulation. This improved modeling is demonstrated by significantly increased follicle diameter and ovulation rates, without a decline in survival, meiotic progression, or spindle morphology (Figure 6). Additionally, the design also allows for easy media changes, imaging, and downstream histological analysis without having to transfer follicles from the agarose mold.

Lastly, timelapse imaging and morphokinetic analysis have already found extensive use in embryo culture for *in vitro* fertilization^84, 85^ and give real-time information on follicle morphokinetics throughout folliculogenesis. Given the ability to perform timelapse imaging and OCT on an individual follicle level without extra steps or manipulations, key morphokinetic characteristics can easily be analyzed to better understand critical parameters needed to support healthy follicle growth. For example, it is known that follicle size is an important marker for oocyte quality in *in vitro* culture systems^68^. Timelapse imaging now provides the ability to determine the exact time when follicles reach maximum growth and perform *in vitro* ovulation to produce a higher quality and mature gamete capable of fertilization, which could be important for IVG as well. Additional optimizations that build on the current concept can be done to further increase experimental output. First, moving to a 96-well culture format has been explored, but further testing will be required to establish this method to culture earlier-stage follicles. Currently, timelapse imaging is limited to one well per plate, but multiple open source and commercial incubator microscopes, allowing for XYZ imaging can be incorporated so that a full culture plate can be imaged^86^.

This method lowers the technical barrier to produce and apply custom agarose molds but has some limitations. First, stereolithography has a limited printing resolution compared to other methods, such as soft lithography^87^, a common microfabrication method performed in a clean room, which further reduces the risk of introducing defects in the master mold. Despite the increased affordability of 3D SLA printers, they still present a large upfront investment. This can be mitigated by using equipment at university core services, partnering with collaborators, or taking advantage of commercial solutions to produce a small number of master molds. Subsequent manufacturing steps are extremely affordable, require no specialized equipment, and can be applied broadly. Further, despite not having observed any significant inter-operator variability, agarose is a natural hydrogel subject to lot-to-lot variability, which could limit reproducibility. Agarose is a widely used and highly biocompatible material, and modulating its concentration will affect the rigidity of the microenvironment and pore size, which could alter the diffusion behavior of molecules^88^. It is also an inert material, lacking peptide sequences for cell adhesion or ECM remodeling. Hence, if a dynamic microenvironment is required, biofunctionalized PEG hydrogels could be used^39^.

The development of novel methods that better mimic *in vivo* conditions is needed to improve current follicle culture techniques. The method of multilayer secondary follicle culture presented here yields similar or improved outcomes compared to established eIVFG methods while being less technically demanding, higher throughput, and allowing for automated analysis. Agarose scaffold-free culture could be expanded towards IVG, aiding basic oocyte biology research, and towards broader goals in assisted reproductive technology, fertility preservation, and species conservation.

## Supplementary Material

Supplementary File 1 Supplementary File 1: CAD file of 24-well master mold base design.

Supplementary File 2 Supplementary File 2: CAD file of 24-well silicone cast container 1.

Supplementary File 3 Supplementary File 3: CAD file of 24-well silicone cast container 2.

Supplementary File 4 Supplementary File 4: CAD file of 96-well master mold base design.

Supplementary File 5 Supplementary File 5: CAD file of 96-well silicone cast container 1.

Supplementary File 6 Supplementary File 6: CAD file of 96-well silicone cast container 2.

Supplementary Video 1 **Supplemental Video S1: Comparative timelapse of alginate eIVFG and agarose scaffold-free culture during the first 24 h.** The movement of the encapsulated follicles during culture precludes automated timelapse analysis. In contrast, follicles are immobilized in agarose culture, which allows analysis by timelapse imaging. Scale bar = 1,000 μm (left) and 400 μm (right).

Materials List

## Figures and Tables

**Table 1:** Types and purpose of media required for the digestion of ovarian tissue for isolation and culture of follicles, *in vitro* maturation of oocytes, and luteinization.

Media	Purpose
Dissection Media (DM)	Isolate ovaries and separate them from the ovarian bursa, oviduct, and periovarian adipose tissue
Enzymatic Media (EM)	Break down ovarian tissue to release ovarian follicles from the stroma
Maintenance Media (MM)	Allow follicles to recover after isolation prior to encapsulation or seeding
Growth Media (GM)	Provide necessary nutrients and signaling molecules that support oocyte growth and development as well as granulosa cell proliferation that further supports the oocyte throughout the culture period.
IVM Media (MatM)	Once follicles have been cultured for 8 days an antrum forms and oocytes grow but are arrested at Prophase of Meiosis I. IVM media mimics the LH surge necessary for ovulation and meiotic progression allows for germinal vesicle breakdown and extrusion of a polar body, indicating that the egg is now arrested in Meiosis II (MII) and ready for fertilization.
Luteinization Media (LM)	After ovulation, granulosa and theca cells luteinize to form a corpora lutea (CL), which produces progesterone and supports early-stage pregnancy if an egg is fertilized and the subsequent embryo implants in the uterus. Luteinization media is utilized to recapitulate this process in vitro.

**Table 2:** Automated tissue processing steps utilizing standard processing protocols.

Step	Solution	Duration
1	70% Ethanol	1 h
2	80% Ethanol	1 h
3	95% Ethanol	1 h
4	95% Ethanol	1 h
5	100% Ethanol	1 h
6	100% Ethanol	1 h
7	100% Ethanol	1 h
8	Xylene	1 h
9	Xylene	1 h
10	Xylene	1 h
11	Paraffin	1 h
12	Paraffin	1 h
