# Supplementary material for: Production and Use of Customizable Agarose Molds for Scaffold-Free Mouse Ovarian Follicle Culture
Source: J Vis Exp. Author manuscript; Available in PMC 2025 Dec 9. (PMC12688028; doi:10.3791/68871)
Supplement: Materials List [file NIHMS2122470-supplement-Materials_List.pdf]

Name of Material/ Equipment	Company	Catalog Number	Comments/Description
**Materials and Chemicals**			
50 mL Polystyrene Centrifuge Tubes	Globe Scientific	941-11017-CS	
1.5 mL Microcentrifuge Tubes	Fisher Scientific	MCT-150	To snap freeze and store media
100 mm Dish, Non-Treated	Fisher Scientific	08-757-100D	To make and sstore agarose micromolds
100% Ethanol	Mercedes Scientific	1200	Tissue Processing
1000 µL Pipette Tips, Low Retention	MIDSCI	PR-1000BK	Culture tips
200 µL Pipette Tips, Low Retention	MIDSCI	PR-200BK	Culture tips
24-well plate	Corning	353047	Culture
35 mm dishes	Falcon	351008	To isolate ovaries and incubate in enyzmatic media
95% Ethanol	Mercedes Scientific	1210	Tissue Processing
Agarose	Hoefer	GR140	Micromolds
BioXtra Mineral Oil, Light Oil, Suitable for mouse embryo cell culture	Sigma-Aldrich	M5310-500ML	Used as a biocompatible demolding agent
Bovine Serum Albumin	MP Biomedicals	103700	Growth Media
Cleaning Dusters, 10 Oz., Pack Of 6	Office Depot	110284	Compressed air
Clear V4 Resin 1 L	Formlabs, Inc	RS-F2-GPCL-04	3D printing resin
Corning® Reusable Plastic Low Form 100 mL Beaker, Polypropylene, Graduated	Corning	1000P-100	Beaker for mixing silicone components
DNAse I	Qiagen	79254	Enzymatic Media
Ecoflex 00-45 Near Clear	Smooth-On, Inc	B09M8Y9PTV	Silicone material
F-12 + GlutaMAX	Gibco	31765-035	Growth Media
Fetal Bovine Fetuin	Sigma	F-3385	Growth Media
Fetal Bovine Serum (FBS)	Peak Serum	PS-FB2	Dissection Media, Enzymatic Media, Maintenance Media & Maturation Media
Fisherbrand™ Sterilization Pouches	Fisher Scientific	01-812-50	Sterilization Pouches

Follicle stimulating hormone (FSH)	EMD Serono, Inc.	Gonal-F Rx only	Growth Media & Maturation Media
Gibco™ DPBS, calcium, magnesium	Fisher Scientific	14-040-182	Dulbecco's phosphate-buffered saline
Human chorionic gonadotropin (hCG)	Sigma Aldrich	C1063-1VL	Maturation Media
Insulin-transferrin-selenium (ITS)	ThermoFisher	41400045	Growth Media
Invitrogen™ Molecular Probes™ Rhodamine Phalloidin	Fisher Scientific	R415	Egg Cytoskeleton Stain
IVF Dishes	Thermo Scientific	150260	To isolate, select for, and pre-equilibrate follicles
Leibovit'z 15 (L15)	Gibco	11415-064	Dissection Media & Enzymatic Media
Liberase	Sigma Aldrich	5401119001	Enzymatic Media
MEMα + GlutaMAX	Gibco	32561-037	Maintenance Media, Growth Media & Maturation Media
Mouse epidermal growth factor (EGF)	BD Biosciences	354010	Maturation Media
Nalgene™ Transparent Polycarbonate Classic Design Desiccator	Thermo Scientific	5311-0250	Vacuum Desiccator
Paraffin	Fisher Scientific	83-30	Tissue Processing & Embedding
Penicillin-streptomycin	Gibco	15140-122	Dissection Media, Enzymatic Media & Maintenance Media
Stripper tips	Origio	MXL3-200	Culture
VECTASHIELD PLUS Antifade Mounting Medium with DAPI	Vector Laboratories	H-2000	Egg nucleus stain
Xylene	Mercedes Scientific	9840	Tissue Processing
α-Tubulin (11H10) Rabbit mAb (Alexa Fluor® 488 Conjugate)	Cell Signaling Technology	5063S	Egg Tubulin stain
**Equipment**			
Build Platform 2	Formlabs, Inc	BP-F3-02-01	3D printer component
Dino-Lite Edge AF4915ZTL, USB 2.0	Dino-Lite	AF4915ZTL	Imager for timelapse setup
Form 3 Resin Tank V2.1	Formlabs, Inc	RT-F3-02-01	3D printer component
Form 3B+ 3D Printer	Formlabs, Inc	F3B-P-PRINTER	Formlabs' stereolithography (SLA) 3D printer
Form cure	Formlabs, Inc	FH-CU-01	Curing platform

Form wash	Formlabs, Inc	FH-WA-01	Washing platform
Spray bottle w/ pump vaporizer	VWR	0309-3005	To apply demolding agent
Light focuser/concentrator cap for Edge series (excluding T5, T8 models)	Dino-Lite	N3C-R	Imager component for timelapse setup
Mount Holder bundle combining the RK-10 rack and RK-10-EX Arm Extension	Dino-Lite	RK-10A	Imager component for timelapse setup
Tissue Embedder	Leica	HistoCore Arcadia	To embed micromolds
Tissue Processor	Leica	TP1020	To process micromolds
211DS Shaking Incubator (Orbital Shaker)	Labnet International	I-5211-DS	To incubate ovaries in enzymatic media for release of follicles
**Software**			
Autodesk Fusion	Autodesk, Inc		Computer-aided design (CAD) software. Autodesk offers free educational access through the Autodesk Education program.
DinoCapture 3.0 version 1.1.1.3	Dino-Lite		
PreForm	Formlabs, Inc		Free print preparation software designed to work with Formlabs' stereolithography (SLA) printers.
ImageJ	National Institutes of Health		To analyze OCT images and measure follicles
